# The road to restore male fertility using *in vitro*-derived germ cells

**DOI:** 10.1093/humupd/dmaf029

**Published:** 2025-12-19

**Authors:** Tiago Macedo, João Pedro Alves-Lopes

**Affiliations:** Department of Women’s and Children’s Health, Karolinska Institutet and Karolinska University Hospital, Stockholm, Sweden; Department of Women’s and Children’s Health, Karolinska Institutet and Karolinska University Hospital, Stockholm, Sweden

**Keywords:** *in vitro*-derived germ cells, germline development, *in vitro* gametogenesis, human pluripotent stem cells, male infertility, cancer long-term side effects

## Abstract

**BACKGROUND:**

Human-induced pluripotent stem cells (hiPSCs) offer immense potential in reproductive medicine, particularly for males who lack germ cells and cannot achieve biological parenthood through conventional ARTs. Early efforts to derive human germ cells from stem cells were hindered by low efficiency, subpar characterization, and the lack of standardized differentiation approaches. However, recent advancements have led to the development of defined protocols that mimic early embryonic development and allow the specification of transcriptomically and epigenetically validated human primordial germ cell-like cells (hPGCLCs). Current research focuses on maturing hPGCLCs *in vitro*, particularly within 3D culture systems that resemble their physiological microenvironment, with the aim of producing transplantable hiPSC-derived spermatogonial stem cells (SSCs) or differentiating them to sperm. At the same time, researchers are also testing whether hiPSCs generated from infertile patients can resume germline differentiation.

**OBJECTIVE AND RATIONALE:**

This narrative review aimed to summarize the key efforts and remaining challenges in differentiating male germ cells from human pluripotent stem cells (hPSCs), with a particular focus on defined and validated protocols for hPGCLC specification. In parallel, we addressed key safety and ethical considerations that must be accounted for the development of clinical applications. A deeper understanding of the approaching therapeutic use of hiPSCs in reproductive medicine is essential for developing novel regenerative fertility strategies.

**SEARCH METHODS:**

PubMed, Scopus, and Web of Science were searched for studies attempting germ cell differentiation from hPSCs using relevant keywords (‘stem cells’, ‘human pluripotent stem cells’, ‘human embryonic stem cells’, ‘human induced pluripotent stem cells’, ‘somatic cell reprogramming’, ‘infertility’, ‘germline’, ‘spermatogenesis’, ‘germ cells’, and ‘primordial germ cells’). No time period restriction was established. Studies with an exclusive focus on female germline differentiation were excluded. To maintain a human-focused perspective, only key animal studies are presented.

**OUTCOMES:**

The literature reveals a clear segregation among protocols for deriving germ cells from hPSCs, particularly between earlier studies lacking standardized differentiation conditions and characterization, and the most recent, defined protocols having transcriptomic and epigenetic validation against *in vivo* hPGCs. Moreover, during the last decade, the field has seen remarkable progress, with multiple efforts aimed at maturing hPGCLCs, closely recapitulating late male embryonic germline development. Additionally, hiPSCs derived from male patients at risk of infertility, particularly those without underlying genetic syndromes, generally retain the capacity for early germline commitment. While attempts at maturating patient germ cells beyond the hPGCLC state remain limited, the rapid pace of discovery and refinement in recent years suggests that further breakthroughs, including clinically applicable fertility restoration strategies, are likely to be achieved in the near future.

**WIDER IMPLICATIONS:**

The ability to generate hiPSCs from infertile patients and to specify them into hPGCLCs supports the feasibility of obtaining hiPSC-derived SSCs for future therapeutic use. These advances raise important ethical, regulatory, and societal questions that must be actively discussed among researchers, clinicians, policymakers, and the general public to ensure responsible and equitable access to these technologies.

**REGISTRATION NUMBER:**

N/A.

## GRAPHICAL ABSTRACT

**Figure:**
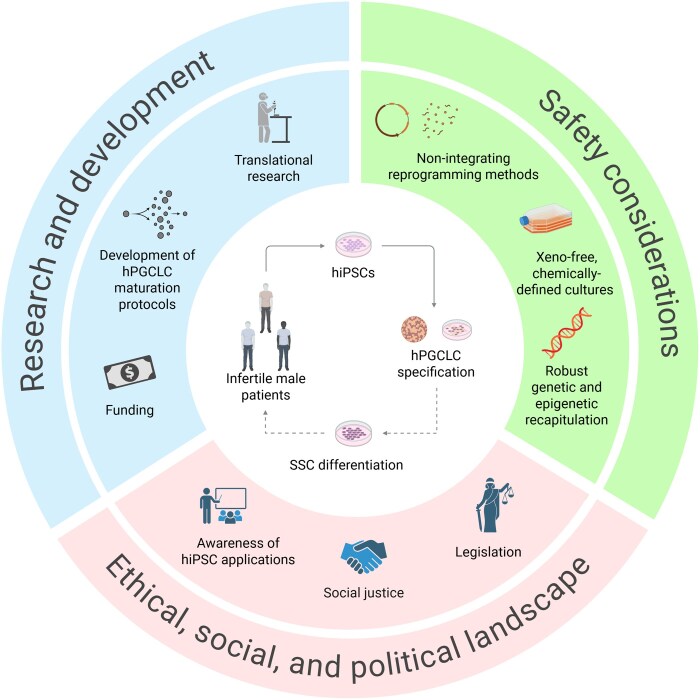
**Recent advances in generating germ cells from patient-derived hiPSCs emphasize the research, safety standards, and ethical frameworks needed for clinical translation.** Figure created using BioRender (https://BioRender.com/k60exa8).

## Introduction

The emergence of human stem cell-based technologies has redefined research, as it holds significant potential for applications in fields such as disease modelling, drug screening, and regenerative medicine (Zakrzewski *et al.*, 2019). Pioneering studies in stem cell research were performed with human embryonic stem cells (hESCs) derived from the inner cell mass of surplus blastocyst embryos generated for assisted reproduction treatments (Thomson *et al.*, 1998). As such, eventual clinical applications were not only ethically controversial but also limited by potential immunogenic concerns (Wert and Mummery, 2003).

Therefore, the introduction of human-induced pluripotent stem cells (hiPSCs), which hold the same potential to resume differentiation into all cellular lineages, marked a significant milestone in stem cell research. Patient-specific hiPSCs can potentially be derived from any adult somatic cell type, thus rendering their applications accessible to a larger number of patients and easier to scale. Takahashi *et al.* were the first to report the successful reprogramming of human somatic cells into hiPSCs based on the overexpression of four transcription factors (POU5F1, SOX2, KLF4, and MYC) shown to orchestrate the cell pluripotency programme (Takahashi *et al.*, 2007).

While early protocols for hiPSC generation heavily relied on the viral delivery and genome integration of reprogramming factors, several safer and simpler alternative approaches have since been developed. These include non-integrating vectors such as mRNA and episomal plasmids, as well as entirely small-molecule chemical-based reprogramming protocols (Cerneckis *et al.*, 2024). One of the main concerns stemming from hiPSC generation is the incomplete reconstitution of the epigenetic reprogramming, with persistent residual somatic signatures in hiPSCs potentially impairing subsequent differentiation efforts (Kim *et al.*, 2010). Nonetheless, the advances in stem cell pluripotency culture conditions have enabled the development of strategies to ‘reset’ residual somatic epigenetic signatures (Buckberry *et al.*, 2023). Overall, this has allowed hiPSCs to be established as a suitable alternative that addresses the ethical, practical, and immunogenic limitations of hESCs while enabling broad clinical accessibility.

Currently, several clinical trials and interventional technologies are underway utilizing hiPSCs to address a variety of disorders, ranging from cardiovascular conditions to neoplasms, with generally favourable outcomes anticipated (Kobold *et al.*, 2023; Lyu *et al.*, 2024). As a rapidly developing field, there is also growing interest in extending their use to other health conditions, such as male infertility.

### The potential of hiPSCs in addressing male infertility

Globally, infertility affects up to 15% of couples, with males estimated to contribute to around half of the cases (Eisenberg *et al.*, 2023). Male infertility remains a poorly understood condition caused by several factors that mainly affect spermatogenesis (Punab *et al.*, 2017). Central to this process are spermatogonial stem cells (SSCs), which constitute the most undifferentiated germ cell state in the adult testis and bear two key properties: the ability to self-renew, ensuring a continuous pool of stem germ cells, and the capacity to differentiate into spermatozoa (Diao *et al.*, 2022). Upon puberty, the maturation of Sertoli and Leydig cells allows the establishment of the necessary niche conditions to trigger SSC differentiation into fully mature spermatozoa (Cheng *et al.*, 2022).

Concerningly, there has been a global 50% reduction in sperm counts over the past five decades (Levine *et al.*, 2023), which is admitted as one of the factors contributing to the decline in birth rates in more developed regions of the world (Fauser *et al.*, 2024). Despite being the most evident, sperm count constitutes only one of several parameters that may cause male infertility, with the WHO urging for equally assessing sperm quality (World Health Organization, 2021). The lack of standardized testing makes it difficult to determine how sperm quality has changed over time, though some studies report decreased motility, increased morphological abnormalities, and rising epigenetic irregularities (Feferkorn *et al.*, 2022; Garcia-Grau *et al.*, 2022; Lismer and Kimmins, 2023; Punjani *et al.*, 2023).

Clinically available ARTs can help to address cases of male infertility where the majority of the ejaculated spermatozoa are not fertilization competent. It is estimated that 3% of births across Europe occur from ART, reaching up to 7–9% in some member states (The European IVF Monitoring Consortium for the European Society of Human Reproduction and Embryology *et al.*, 2023). However, ART is not a viable alternative when very few to no viable germ cells remain in the testes. Among this subset of the male population are patients with genetic abnormalities. These account for 15% of male infertility cases, with the most common cause of complete sperm production failure being chromosomal anomalies such as Klinefelter syndrome (Krausz and Riera-Escamilla, 2018).

Likewise, cancer patients represent a population of males at risk of losing their germ cells (Izurieta-Pacheco *et al.*, 2025). This outcome is largely attributed to treatment regimens with high doses of alkylating agents and/or radiotherapy (Green *et al.*, 2014; Vern-Gross *et al.*, 2015). As such, gonadotoxic risk stratifications of the most common regimens (Poorvu *et al.*, 2019) allow clinicians to preemptively suggest to adult patients the possibility of sperm cryopreservation. This is a standard practice in many medical centres across the globe, but it cannot be offered to prepubertal childhood cancer patients lacking functional sperm. In such cases, the approaches for fertility preservation remain experimental and not widely available (Goossens *et al.*, 2020).

Currently, the most common practice for prepubertal patients is the cryopreservation of testicular tissue samples prior to treatment. It is estimated that over 3000 prepubertal boys have already undergone this procedure throughout the world (Duffin *et al.*, 2024). Subsequent clinical attempts to restore fertility have relied on the existence of viable SSCs in the preserved tissue, which can be injected back into the patient’s testes after being isolated and expanded (Zielen *et al.*, 2025) or auto-transplanted within the supportive testicular niche as a tissue graft (Jensen *et al.*, 2024). Despite these earlier unsuccessful efforts in restoring spermatogenesis, investigations are progressing to optimize and standardize existing protocols. In this regard, additional ethical approvals for pilot studies have already been granted (Duffin *et al.*, 2024).

As an alternative, cryopreserved SSCs could be entirely differentiated *in vitro* up to a fertilization-competent stage and later used in ART techniques to generate offspring (Alves-Lopes and Stukenborg, 2018; Oliver and Stukenborg, 2020). In this context, SSCs from cryopreserved immature testicular tissue were previously reported to differentiate up to haploid spermatid-like cells, both within tissue cultures (de Michele *et al.*, 2018) and upon isolation and self-reorganization in a 3D organoid system (Galdon *et al.*, 2024), though further optimization to increase the efficiency of the process and a comprehensive validation of the obtained spermatid-like cells are still required.

While long-term cryopreservation does not seem to impact spermatogonial viability (Wyns *et al.*, 2008; Portela *et al.*, 2019), patients may already lack these cells when the tissue is collected, thus rendering ART approaches unfeasible. Indeed, a recent report revealed that roughly half of the testicular samples collected from prepubertal cancer patients treated with alkylating agents had spermatogonia numbers below the defined critical threshold (Funke *et al.*, 2021). It is also known that this concern extends beyond the growing population of cancer survivors and includes patients with other potential infertility-causing conditions, such as genetic syndromes and haematological diseases (Stukenborg *et al.*, 2018; Funke *et al.*, 2021; Lahtinen *et al.*, 2024). Moreover, as previously suggested, even if some SSCs remain through to adulthood, their functionality can be significantly impaired due to those conditions and their respective treatments (Ribas-Maynou *et al.*, 2021). Notably, upon evaluation of the prepubertal testicular samples stored within a network of medical centres across the world, it was found that around 40% had been collected after the start of gonadotoxic treatments (Valli-Pulaski *et al.*, 2019).

Given the strong preference for biological parenthood (Rulli, 2016; Hendriks *et al.*, 2017), there is an urgent need to develop alternative techniques for deriving patient-specific germ cells. If physiological human SSCs bear the capacity to repopulate the testis and resume spermatogenesis, as demonstrated for mice and rhesus monkeys (Hermann *et al.*, 2012; Serrano *et al.*, 2021; Shetty *et al.*, 2021), achieving similarly competent hiPSC-derived SSCs represents the most promising solution to regenerate fertility in individuals with a deprived pool of endogenous SSCs, even considering the ability to transpose recent findings on ‘mitomeiosis’ to male gametes (Marti Gutierrez *et al.*, 2025). In addition to this, hiPSC-derived SSCs may be used as the starting population in the efforts to reconstitute the full spermatogenesis process *in vitro* (Robinson *et al.*, 2023) and obtain hiPSC-derived sperm, ensuring an alternative for fertility restoration.

Accordingly, this strategy involves three key tasks: (i) derivation of hiPSC lines from infertile patients or those at risk of infertility, (ii) differentiation of hiPSCs to the SSC state, and (iii) auto-transplantation of patient-specific hiPSC-derived SSCs back into their testes. This roadmap strategy was already kick-started with the ability to reconstitute early embryonic gametogenesis events from hiPSCs, namely the specification of human primordial germ cell-like cells (hPGCLCs), the *in vitro* counterpart of human primordial germ cells (hPGCs) (Irie *et al.*, 2015; Sasaki *et al.*, 2015).

Human PGCs constitute the first germline-committed cell type in human embryos, specified at around Weeks 2–3 after fertilization (Tang *et al.*, 2016; Hargy and Sasaki, 2023). Whilst migrating towards the genital ridge during Weeks 3–5, hPGCs start an extensive epigenetic reprogramming, including an impressive global DNA demethylation process (Ramakrishna *et al.*, 2025). Upon colonization of the developing gonads, during Weeks 5–6, hPGCs continue undergoing complex transcriptomic and epigenetic maturation processes (Tang *et al.*, 2015; Garcia-Alonso *et al.*, 2022; Gruhn *et al.*, 2023), culminating with the differentiation into pro-spermatogonia, during Weeks 8–10, in the developing testis. Subsequently, pro-spermatogonia mature into SSCs during late foetal and early postnatal life. After puberty and during adulthood, SSCs start complex processes of self-renewal and differentiation that simultaneously maintain their numbers and lead to the production of sperm through the process of spermatogenesis (Saitou and Hayashi, 2021).

Due to the limited access to human embryos at early stages of development, a comprehensive characterization of the pathways that orchestrate both hPGC specification and early maturation have remained elusive for a long time (Pereira Daoud *et al.*, 2020). Nevertheless, recent *ex vivo* studies on rare early human embryos (Tyser *et al.*, 2021; Xiao *et al.*, 2024; Cui *et al.*, 2025), along with *in vitro* protocols to model early embryonic events from human pluripotent stem cells (hPSCs), including specification of hPGCLCs, brought the opportunity to uncover some of the mechanisms underlying the specification and maturation of this lineage (Saitou and Hayashi, 2021).

A pivotal development in this context has been the growing body of research on hPSC pluripotency, which led to the identification of optimal culture conditions that enable both the conversion and maintenance of hPSCs in the ground state of naïve pluripotency (Zhou *et al.*, 2023). Naïve hPSCs mirror the transcriptomic and epigenetic profiles of the pre-implantation inner cell mass population (Takashima *et al.*, 2014; Theunissen *et al.*, 2014; Guo *et al.*, 2016), capturing the most developmentally unrestricted state of pluripotent stem cells.

To date, efforts to obtain hiPSC-derived male germ cells have successfully recapitulated the embryonic stages of gametogenesis, progressing up to pro-spermatogonia-like cells (Hwang *et al.*, 2020; Murase *et al.*, 2024). In this review, we present an overview of the attempts at male germ cell differentiation from hPSCs, narrowing our focus to studies that have achieved the state of transcriptomically validated hPGCLCs and their further maturation *in vitro*. Furthermore, we compiled studies that describe the generation of hiPSCs from infertile males or individuals at risk of infertility, along with their subsequent differentiation into the germline fate. Finally, given the rapid development of the field in the past decade, we discuss the need to establish normative frameworks encompassing ethical, social, political, and safety considerations to guide the clinical translation of hiPSC-based technologies for male fertility restoration.

## Methods

A literature search was conducted in PubMed, Scopus, and Web of Science to identify studies on germ cell differentiation from hPSCs using combinations of the following key words: ‘stem cells’, ‘human pluripotent stem cells’, ‘human embryonic stem cells’, ‘human induced pluripotent stem cells’, ‘somatic cell reprogramming’, ‘infertility’, ‘germline’, ‘spermatogenesis’, ‘germ cells’, and ‘primordial germ cells’. No time restrictions were applied. Studies exclusively addressing female germline differentiation were excluded, and only essential animal studies were retained to provide essential context for human findings.

## Advances in *in vitro* human germ cell differentiation from pluripotent stem cells

Prior to the emergence of comprehensive transcriptomic techniques and protocols for the isolation and characterization of gonadal hPGCs, methods for deriving germ cells from hPSCs lacked thorough validation of cell identity. For instance, the sole reliance on the expression of a few markers, some of which are currently known not to be specific to early germ cells, raises some doubts about the identity of the obtained cells. Moreover, these early efforts often resulted in low specification efficiencies (<20%), poorly defined differentiation processes, and limited reproducibility.

Nevertheless, we believe it is relevant to cover the historical context of the pioneer approaches to germ cell differentiation, which we have designated as ‘undefined’ and expanded upon in the section below. Conversely, in 2015, two independent publications first reported ‘defined’ protocols to generate transcriptomically validated hPGCLCs (Irie *et al.*, 2015; Sasaki *et al.*, 2015). These breakthroughs propelled recent attempts at reconstituting the human male germline to adopt a more standardized two-step process: (i) specification and isolation of hPGCLCs and (ii) maturation of hPGCLCs into more advanced germ cell states, such as pro-spermatogonia-like cells. This progress has also elevated expectations in the field, emphasizing the need for rigorous epigenetic validation to ensure developmental fidelity.

### Undefined differentiation of germ cells from hPSCs

Clark *et al.* pioneered the field by first reporting spontaneous germ cell differentiation from hPSCs in embryoid bodies (EBs; 3D aggregates of differentiating pluripotent stem cells), identified based on the expression of germ cell markers such as *GDF9* and *TEKT1* (Clark *et al.*, 2004). This work sparked subsequent efforts to characterize the signalling pathways driving germline differentiation and to identify surface markers to isolate this cell population.

A key finding of this pursuit came from the validation that bone morphogenic proteins (BMPs) enhance germ cell differentiation from hPSCs, as previously observed in murine models (Lawson *et al.*, 1999; Ying *et al.*, 2001). Indeed, Kee *et al.* (2006) observed that supplementing the culture media with BMP proteins led to a significant increase in germ cell markers after 3 days in culture, especially at the outermost layers of the EBs.

At this stage, there was a marked interest in isolating *in vitro*-derived germ cells for downstream analyses and expanding them *in vitro* (West *et al.*, 2008). However, the lack of suitable cell-surface markers was one of the main obstacles in these efforts. As such, it was relevant to explore whether PGC cell-surface markers described for other species were conserved in humans.

Using a monolayer specification system, Bucay *et al.* (2009) proposed CXCR4 as a novel *in vitro*-derived germ cell-surface marker. This receptor protein was shown to be expressed by migratory mouse PGCs in hindgut organ cultures, establishing a ligand–receptor interaction with the SDF1 protein, which is crucial for guiding mouse PGCs towards the developing gonads (Molyneaux *et al.*, 2003). However, its relevance in human PGC migration remains unknown. Nevertheless, the authors reported the emergence of CXCR4+ cells co-expressing *PRDM1* and *KIT* within 7 days of culture, revealing the potential of CXCR4 as a human germ cell marker candidate. Meanwhile, CXCR4 was also reported to be expressed in early mesendoderm (Nelson *et al.*, 2008) and neural embryonic lineages (Zhang *et al.*, 2016), compromising its reliability as a single marker for germ cell isolation.

Similarly, migratory mouse PGCs were reported to express EPCAM (Anderson *et al.*, 1999). Starting from hESCs, it was possible to obtain double-positive POU5F1/EPCAM cells after a 4-day differentiation in EBs, with the subsequent seeding on 2D gelatin-coated dishes for up to 30 days. Moreover, the yield of POU5F1/EPCAM double-positive cells was up to 10 times higher after BMP4 and WNT3A supplementation (Chuang *et al.*, 2012).

At around the same time, the emergence of hiPSC reprogramming protocols from somatic cells (Takahashi *et al.*, 2007) marked a progressive shift from the use of hESCs towards hiPSCs as precursors for *in vitro*-derived germ cells. Still relying on non-standardized culture conditions and markers, the differentiation efficacy was found to be comparable to the reports utilizing hESCs, further highlighting the relevance of hiPSCs in reproductive medicine.

Park *et al.* (2009) first reported that hiPSCs, while not derived from the inner cell mass of blastocysts like hESCs, still bear the potential for germline fate. In a monolayer differentiation system on inactivated human gonadal stromal cells, both hESCs and hiPSCs derived from foetal skin fibroblasts gave rise to presumable KIT+/SSEA1+ germ cells within 7 days.

Later, utilizing a 14-day BMP-supplemented monolayer culture, Panula *et al.* reported that hiPSCs derived from adult dermal fibroblasts could also give rise to *in vitro*-derived germ cells expressing *NR6A1*, *IFITM1*, *DPPA3*, and *PELO*. Moreover, overexpression of *DAZL*, *BOLL*, and *DAZAP1* was reported to facilitate meiosis entry (Panula *et al.*, 2011). Likewise, a similar approach later showed that overexpressing *DDX4* promoted progression through meiosis at Day 14, though not with a synergistic effect when combined with the overexpression of *DAZL* (Medrano *et al.*, 2012).

Lastly, Easley *et al.* (2012) attempted to generate germ cells from hiPSCs using a defined lipid-rich medium that was previously shown to sustain the proliferation of mouse SSCs for several months and even retain their differentiation capability (Kanatsu-Shinohara *et al.*, 2003). The cells obtained after 10 days were reported to express a few early germline and even SSC markers.

Direct differentiation of hiPSCs into SSCs without transitioning through the hPGC states, as reported in the previous and more contemporary approaches (Eguizabal *et al.*, 2011), could offer an efficient platform for downstream applications. However, an *in vitro* stepwise reconstitution of embryonic and foetal developmental events, especially the key epigenetic reprogramming features, seems indispensable to reliably achieve germline states with an *in vivo*-like profile and functionality.

Indeed, while the previously described attempts provided important contributions and embodied the notion that differentiating hiPSCs into germ cells is an achievable goal, there were key limitations in these protocols. As mentioned before, the reliance on a restricted number of hPGC markers to confirm the presence of nascent hPGCLCs raises some concerns regarding the identity of the germ cells that were obtained, with the transcriptomic and epigenomic profiles not validated against their *in vivo* counterparts. Furthermore, the yields of these *in vitro*-derived germ cells were consistently low (<20%).

Parallel to these efforts, the realm of the mouse model was significantly more advanced, achieving the successful birth of offspring from *in vitro*-derived germ cells as early as 2011 (Hayashi *et al.*, 2011, 2012). As such, this urged researchers to translate the main takeaways to the human context. One crucial notion for the success of the mouse model was the realization that PGCLC specification became significantly more efficient when stem cells were first induced to epiblast-like cells (EpiLCs), an intermediate state between naïve and primed pluripotency (Hayashi *et al.*, 2011).

In turn, the first cultured hPSCs shared unique properties with mouse PSCs maintained in the primed state of pluripotency, which had limited germline differentiation potential (Brons *et al.*, 2007). Having established this concept, researchers started to develop strategies to convert hPSCs into transient cell states capable of efficiently specifying the hPGC fate, aiming for a breakthrough in developmental biology and reproductive medicine.

### Defined hPGCLC specification from hPSCs

**Figure 1.**
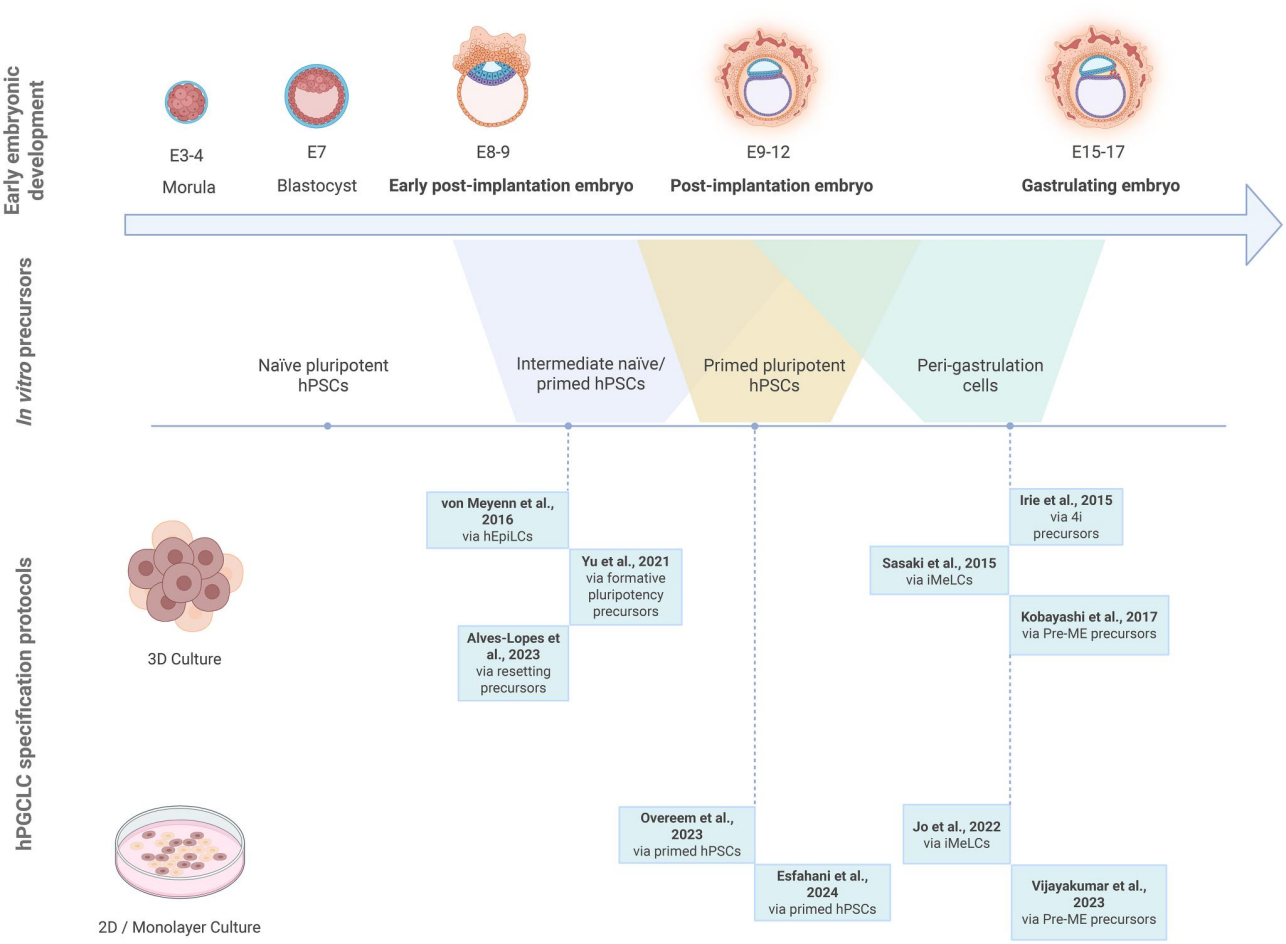
**Overview of defined hPGCLC specification protocols.** The upper section illustrates the timeline of early human embryonic development, spanning approximately from embryonic Day 3 (E3) to E17. The middle panel represents the spectrum of *in vitro* pluripotency, highlighting the known competence states for hPGCLC specification: (i) intermediate naïve/primed hPSCs; (ii) primed hPSCs, and (iii) peri-gastrulation cells and their corresponding *in vivo* epiblast counterparts. The lower section presents the currently established defined protocols for specifying hPGCLCs from each of these precursor states, categorized into 3D and monolayer approaches. hPSCs, human pluripotent stem cells; hPGCLCs, human primordial germ cell-like cells; 4i, four inhibitors culture; iMeLCs, incipient mesoderm-like cells; hEpiLCs, human epiblast-like cells; Pre-ME, pre-mesendoderm precursors. Figure created using BioRender (https://BioRender.com/agvyb08).

To address the limited characterization of the germ cell fraction obtained from the differentiation of hPSCs, recent protocols built upon defined descriptions of hPGCLC specification and included additional layers of comprehensive transcriptomic and epigenetic validation against their *in vivo* counterparts. These approaches follow the strategy of first inducing primed hPSCs into either peri-gastrulation (representing epiblast cells at the onset of gastrulation) (Irie *et al.*, 2015; Sasaki *et al.*, 2015; Kobayashi *et al.*, 2017) or into primed/naïve intermediary state precursors (von Meyenn *et al.*, 2016; Yu *et al.*, 2021; Alves-Lopes *et al.*, 2023) before specifying hPGCLCs. Using standardized culture conditions and specific cell-surface markers to better identify and isolate the germline population, these approaches robustly allow the generation of hPGCLCs with an efficiency of ∼20–40% (Table 1 and Fig. 1).

**Table 1.** Defined hPGCLC specification from hPSCs.

Reference	Cell source	Reprogramming method (for hiPSCs)	hPGCLC specification strategy	hPGCLC markers (identification and/or isolation)	Outcome
Irie *et al.*, 2015	Male and female hESCs	N/A	Via peri-gastrulation precursors (4i)	**FACS:** NANOS3 (reporter), TNAP (cell surface marker)	**hESCs at specification Day 4:** Up to ∼46% hPGCLC induction efficacy.
	Fragile X male hiPSCs (from dermal fibroblasts) Healthy female hiPSCs (from dermal fibroblasts)	Viral vector	Via peri-gastrulation precursors (4i)	**FACS:** TNAP, CD38 (cell surface markers)	**hiPSCs at specification Day 4:** Up to ∼31% hPGCLC induction efficacy.
Sasaki *et al.*, 2015	Male and female hiPSCs (from dermal fibroblasts and peripheral blood cells)	Viral vector Episomal vector	Via primed (StemFit) and peri-gastrulation precursors (iMeLCs)	**FACS:** PRDM1, TFAP2C (double reporter)	**hiPSCs (from primed pluripotency) at specification Days 4–8:** Up to ∼20% hPGCLC induction efficacy. hiPSCs (via iMeLCs) at specification Day 4: Up to ∼60% hPGCLC induction efficacy.
von Meyenn *et al.*, 2016	Male and female hESCs	N/A	Via precursors between naïve and primed pluripotency (hEpiLCs)	**FACS:** KIT (cell surface marker)	**hESCs at specification Day 12:** Up to ∼26% hPGCLC induction efficacy.
Kobayashi *et al.*, 2017	Male and female hESCs	N/A	Via peri-gastrulation precursors (Pre-ME)	**FACS:** NANOS3 (reporter), TNAP (cell surface marker)	**hESCs (via Pre-ME precursors) at specification Day 4:** Up to ∼38% hPGCLC induction efficacy.
Yu *et al.*, 2021	Male hiPSCs (from foreskin fibroblasts)	Episomal vector	Via precursors between naïve and primed pluripotency (formative FTW-hiPSCs)	**FACS:** EpCAM, ITA6 (cell surface markers)	**hiPSCs at specification Day 4:** Up to ∼26% hPGCLC induction efficacy.
Jo *et al.*, 2022	Female hESC Male and female hiPSCs	Viral and episomal vector	Via primed (mTeSR1) and peri-gastrulation (iMeLCs) precursors	**IF:** PRDM, TFAP2C, SOX17, NANOG (intracellular markers)	**hiPSCs at 42 h of specification culture:** Up to ∼70% hPGCLC induction efficacy
Alves-Lopes *et al.*, 2023	Male hESCs	N/A	Via precursors between naïve and primed pluripotency (resetting precursors)	**FACS:** NANOS3 (reporter), TNAP, PDPN (cell surface markers)	**hESCs at specification Day 4:** Up to 35% hPGCLC induction efficacy.
Overeem *et al.*, 2023	Male hESCs hiPSCs (no info available)	N/A	Via primed precursors (mTeSR-Plus)	**FACS:** ITA6, EPCAM (cell surface markers)	**hPSCs at specification Day 5:** Up to 40% hPGCLC induction efficacy.
Vijayakumar *et al.*, 2023	Male hESCs Male hiPSCs (from foreskin fibroblasts)	RNA vector	Via peri-gastrulation precursors (Pre-ME)	**FACS:** NANOS3 (reporter)	**hPSCs at specification Day 3.5:** 20–30% hPGCLC induction efficacy.
Esfahani *et al.*, 2024	Male and female hESCs Male hiPSC (no info)	N/A	Via primed precursors (mTeSR)	**FACS:** TFAP2C, SOX17 (intracellular markers)	**hPSCs at specification Day 8:** Up to ∼20% hPGCLC induction efficacy

hPGCLCs, human primordial germ cell-like cells; hPSCs, human pluripotent stem cells; hESCs, human embryonic stem cells; hiPSCs, human-induced pluripotent stem cells; 4i, four inhibitors culture; iMeLCs, incipient mesoderm-like cells; hEpiLCs, human epiblast-like cells; Pre-ME, pre-mesendoderm precursors; FTW-hiPScs, FGF, TGF-β/Smad, WNT/β-catenin hiPSCs; FACS, fluorescence-activated cell sorting; IF, immunofluorescence; N/A, not applicable.

In 2015, two independent studies described for the first time defined methods to address the poor germline competence of primed hPSCs (Irie *et al.*, 2015; Sasaki *et al.*, 2015). Irie *et al.* used a previously reported medium thought to convert primed hPSCs into naïve hPSCs, the four inhibitors (4i) medium (Gafni *et al.*, 2013). Notably, TNAP+/NANOS3+ hPGCLCs could be specified directly from hPSCs maintained in 4i medium, with an efficiency of ∼46%. Today, it is accepted that hPSCs cultured in 4i medium are closer to the primed/peri-gastrulation state than the naïve state of pluripotency (Bayerl *et al.*, 2021; Alves-Lopes *et al.*, 2023). Importantly, the hPGCLCs specified with this protocol shared a significant fraction of their transcriptional profile with that of gonadal hPGCs from Week 7 male embryos, missing the migratory/gonadal hPGC markers (e.g. DDX4 and DAZL), as expected. These hPGCLCs, further shown to be initiating the DNA demethylation programme, were therefore considered to resemble the nascent/pre-migratory hPGC state. On the mechanistic level, the authors demonstrated that SOX17 was the key orchestrator of hPGCLC specification, which contrasts with mouse PGCLC specification, where SOX2 is the equivalent essential counterpart (Campolo *et al.*, 2013). Additionally, the authors showed that PRDM1 activates the hPGC programme and represses somatic cell fates, as described in the mouse model.

In turn, Sasaki *et al.* (2015) demonstrated that primed hPSCs are capable of directly specifying PRDM1+/TFAP2C+ hPGCLCs when cultured in StemFit medium. This is likely due to StemFit allowing hPSCs to assume a state more in line with the *in vivo* early post-implantation posterior epiblast. Still, the yield was considered relatively low, with many cells dying during the process. As such, the authors developed a strategy to differentiate primed hPSCs to ‘incipient mesoderm-like cells’ (iMeLCs), using Activin A and CHIR99021, a WNT signalling pathway agonist molecule. Notably, starting from this peri-gastrulation precursor state, the specification protocol yielded a much larger fraction of up to ∼60% hPGCLCs, contrasting with the ∼15–20% efficacy of the direct induction from primed hPSCs. When characterizing the sorted hPGCLC fraction, DNA methylation levels were shown to be lower than in the originating hiPSCs. Additionally, the transcriptomic profile was found to highly correlate with that of hPGCLCs obtained by Irie *et al.* (2015). Their transcriptomic analysis also showed that hPSCs maintained in 4i conditions upregulated mesodermal markers, including *EOMES* and *SP5*, indicating a transcriptional profile more closely aligned with iMeLCs (Sasaki *et al.*, 2015).

In addition to the 4i and iMeLC protocols, other independent studies have adopted similar strategies to obtain peri-gastrulation precursors for hPGCLC specification. Kobayashi *et al.* first assessed the window of germline competence by treating primed hPSCs cultured in E8 medium, which are not competent for acquiring the germ cell fate, with Activin A and CHIR99021 to initiate mesendodermal differentiation. Accordingly, a 12 h exposure was ideal in promoting germ cell fate specification, yielding up to 38% NANOS3+/TNAP+ hPGCLCs (Kobayashi *et al.*, 2017). These intermediary state cells were termed pre-mesendoderm (Pre-ME) precursors and described to represent human epiblast cells at the onset of gastrulation.

Parallel to these efforts, the increasing knowledge on modulating the pluripotency states of hPSCs led to the establishment of other types of precursors for hPGCLC specification, with pluripotency profiles between the primed and naïve states (Zhou *et al.*, 2023). Accordingly, von Meyenn *et al.* (2016) started from naïve hPSCs and pre-induced them to human EpiLCs with transforming growth factor -β (TGF-β) and fibroblast growth factor 2 (FGF2), adopting a strategy similar to that described by Hayashi *et al.* (2011) for mouse PGCLC specification. The yield of KIT+ hPGCLCs at Day 12 was around 26%, accompanied by a gradual DNA demethylation over the 12 days in culture. In addition to the co-staining of hPGC markers PRDM1, SOX17, and NANOS3, the obtained population of hPGCLCs also had a close transcriptomic profile to that of *in vivo* hPGCs.

Likewise, Yu *et al.* (2021) investigated the optimal culture conditions for maintaining hPSCs in a pluripotency state reflective of the formative epiblast, an intermediary state proposed to stand between naïve and primed pluripotency (Smith, 2017). Accordingly, they found that hPSCs could be maintained in the formative state of pluripotency when cultured in a media supplemented with FGF2, Activin A, and CHIR99021. Moreover, these precursors could reliably give rise to ∼26% EPCAM+/ITA6+ hPGCLCs after BMP induction. The hPGCLC character was further confirmed by immunofluorescence (IF) staining against TFAP2C, PRDM1, and NANOS3.

With the two previous studies establishing the intermediary state naïve/primed hPSCs as potential hPCGLC precursors, it was still unknown how germline competence varied across the entire spectrum of naïve to primed pluripotency and, just as importantly, how hPGCLCs derived from distinct precursors differed from each other. In this sense, Alves-Lopes *et al.* first investigated the hPGCLC specification competence of transient hPSCs during their conversion from naïve to primed pluripotency, ‘capacitating’ precursors, and vice versa, ‘resetting’ precursors. The authors established that fully reset naïve hPSCs (Guo *et al.*, 2016; Bayerl *et al.*, 2021), like primed hPSCs cultured in E8 conditions, have low potential to generate hPGCLCs (Alves-Lopes *et al.*, 2023). In turn, resetting precursors bearing a transient pluripotency state have significant germline competence, giving rise to up to 25–35% hPGCLCs validated by IF (NANOS3, SOX17, NANOG, POU5F1, and PRDM1), fluorescence-activated cell sorting based on the combination of NANOS3, TNAP, and PDPN markers, and RNA sequencing. Notably, when compared with hPGCLCs obtained from peri-gastrulation precursors (Irie *et al.*, 2015; Kobayashi *et al.*, 2017), resetting hPGCLCs showed a transcriptomic signature characteristic of early migratory hPGCs and an enhanced progression capability, comparable to that observed with *in vivo* hPGCs.

At this stage, the defined generation of transcriptomically validated hPGCLCs had been firmly achieved, though it still relied on considerably laborious and low-throughput specification within EBs. Owing to slightly different parameters involved with hPGCLC induction, including starting precursors, EB size, cytokine concentrations, induction times, and marker selection for isolation, there was still some variability reported in the specification efficiency and hPGCLC profiles. This variability might also be due to inherent line-specific differences, as inducing different hPSC lines under the same conditions was shown to generate heterogeneous yields in terms of hPGCLC specification efficiency (Chen *et al.*, 2017; Yokobayashi *et al.*, 2017). In these studies, it was speculated that differences in hPGCLC specification efficiencies might be related to differences in the epigenetic landscape, but the confirmation of this effect remains to be demonstrated. As such, efforts to develop scalable and more practical monolayer protocols capable of consistently yielding larger amounts of hPGCLCs for downstream applications have resurfaced once again.

In this regard, Vijayakumar *et al.* (2023) adapted the Pre-ME protocol (Kobayashi *et al.*, 2017) to a streamlined 2D approach. By adjusting the time at which BMP, stem cell factor (SCF), and epidermal growth factor (EGF) were supplemented, the authors achieved a NANOS3+ hPGCLC specification efficacy of ∼20–30% on Day 3 of their cultures. While the relative efficiency is comparable to previous EB-based attempts, a monolayer culture allows the generation of hPGCLCs in much higher absolute numbers. These hPGCLCs were shown to have a transcriptomic profile similar to EB-generated hPGCLCs and early hPGCs (Li *et al.*, 2017).

Likewise, Overeem *et al.* (2023) developed a novel monolayer method based on a basement membrane extract overlay, allowing direct specification of hPGCLCs from primed hPSCs. Combining the 2D nature of the culture with the extract’s supportive properties not only significantly reduced the required BMP concentrations but also eliminated the need for an intermediary pre-induction step. Notably, these conditions yielded up to ∼40% ITA6+/EPCAM+ hPGCLCs with a transcriptomic profile that shared similarities to that of physiological hPGCs obtained from a Carnegie Stage 7 embryo (Tyser *et al.*, 2021). Interestingly, cell lines previously shown to inefficiently generate hPGCLCs under other EB-based protocols still exhibited low germline competence, suggesting once again that this may be an inherent cell line property.

In line with this trend, Esfahani *et al.* adapted a previously described flat culture biomimetic system (Shao *et al.*, 2017), also based on the supplementation with a basement membrane extract, to specify hPGCLCs (Esfahani *et al.*, 2024). This system allowed high-throughput hPGCLC specification, critical for downstream applications, such as hPGCLC maturation cultures and hPGCLC competence screening of patient-derived hiPSCs. In addition, specification occurs directly from primed hPSCs without the need for cytokine supplementation, including BMPs. Under these conditions, SOX17+/TFAP2C+ hPGCLCs could be generated with an efficacy of ∼20% within 8 days and demonstrated a high transcriptomic correlation with hPGCs and hPGCLCs obtained from 3D EB protocols (Esfahani *et al.*, 2024).

Additionally, the development of confined 2D micropattern cultures of hPSCs allowed more controlled studies of the signalling dynamics driving gastrulation events (Warmflash *et al.*, 2014). It was found that this setting gave rise to SOX17+ cells upon BMP signalling, which were initially presumed to belong to the endoderm lineage, though they lacked expression of other endoderm markers (Martyn *et al.*, 2019). Aiming to clarify the identity of this cell population, Jo *et al.* (2022) developed a novel image analysis methodology, confirming the identity of those cells as PRDM1+/TFAP2C+/SOX17+/NANOG+ hPGCLCs. Under the micropattern cultures, hPGCLCs can be specified with a remarkable 50% efficacy after optimizing the initial colony size alone and 70% after a pre-induction step to iMeLCs. In addition, these hPGCLCs were shown to bear a transcriptomic profile close to that of hPGCs from a Carnegie Stage 7 embryo (Tyser *et al.*, 2021).

Overall, the work on modelling the physiological specification of hPGCs reviewed in this section, and summarized in Table 1 and Fig. 1, was crucial for understanding some of the developmental dynamics and transcriptional regulation of nascent hPGCs. In the developing embryo, hPGC maturation continues during their migratory route through the hindgut and dorsal mesentery and subsequent colonization of the primitive gonads. Therefore, while the protocols mentioned here allow defined specification of hPGCLCs, these systems do not provide the molecular and spatial cues required for further germline development, including the modulation of the epigenetic resetting process. This highlighted the need for developing novel complementary approaches better suited for hPGCLC maturation, which are discussed in the section below.

### hPGCLC maturation

With defined methods to reliably obtain hPGCLCs from hPSCs now established, the current challenge lies in developing culture conditions that support hPGCLC maturation towards more advanced developmental states. In mice, the generation of fertile offspring from hiPSC-derived gametes was achieved over a decade ago (Hayashi *et al.*, 2011, 2012). Despite this, recapitulating human gametogenesis remains a challenging undertaking. The first successful approach to mature hPGCLCs was achieved through co-culture with mouse gonadal somatic cells, demonstrating the critical importance of the paracrine cues and cell-to-cell support from the embryonic niche in further progressing germline differentiation (Yamashiro *et al.*, 2018; Hwang *et al.*, 2020) (Table 2). However, the low efficiency, long culture period, and reliance on primary mouse cells make this approach incompatible with therapeutic usage. Hence, the development of defined and feeder-free alternatives remains a critical next step for the field.

**Table 2.** Male hPGCLC maturation attempts.

Reference	hPGCLC specification strategy	Starting cell population	Maturation method	Outcome
Yamashiro *et al.*, 2018	Via peri-gastrulation precursors (iMeLCs)	Newly specified Day 6 PRDM1+/TFAP2C+ hPGCLCs	Aggregation with fetal mouse ovarian somatic cells	Expression of DAZL and DDX4 at aggregation Day 77, with an overall transcriptomic profile similar to mitotic gonocytes. Global DNA methylation levels decreased to ∼10%.
Murase *et al.*, 2020	Via peri-gastrulation precursors (iMeLCs)	Expanded Day 30 PRDM1+/TFAP2C+ hPGCLCs	Aggregation with fetal mouse ovarian somatic cells	Higher survival of expanded hPGCLCs relative to newly specified Day 4–6 hPGCLCs upon aggregation with somatic cells. Expression of DAZL and DDX4 starts to be detected at aggregation Day 30. Global DNA methylation levels decreased to ∼10%.
Hwang *et al.*, 2020	Via peri-gastrulation precursors (iMeLCs)	Newly specified Day 5 TFAP2C+ hPGCLCs	Aggregation with fetal mouse testicular somatic cells	hPGCLCs aggregated within tubular structures at aggregation Day 14. Expression of DAZL and DDX4 at aggregation Days 81–120, with an overall transcriptomic profile similar to mitotic prospermatogonia/gonocytes, with some cells reaching the mitotic arrest pro-spermatogonia state.
Kobayashi *et al.*, 2022	Via peri-gastrulation precursors (4i)	Newly specified Day 8 CD38+ hPGCLCs	Monolayer expansion and aggregation with mouse testicular somatic cells	hPGCLCs could be expanded for at least 160 days. Expression of DAZL at aggregation Day 77.
Alves-Lopes *et al.*, 2023	Via peri-gastrulation (4i and Pre-ME) and resetting precursors	Newly specified Day 4 NANOS3+, hPGCLCs	Co-culture of hPGCLCs with human hindgut organoids	hPGCLCs derived from resetting precursors mature at a tempo similar to that observed *in vivo*. Resetting hPGCLCs expressed DAZL (heterogeneously) at co-culture Day 25, while peri-gastrulation hPGCLCs did not.
Murase *et al.*, 2024	Via peri-gastrulation precursors (iMeLCs)	Newly specified Days 6–8 PRDM1+/TFAP2C+, EPCAM+/ITGA6+ hPGCLCs	Molecular-driven hPGCLC differentiation in monolayer cell culture	BMP2 supplementation promoted hPGCLC differentiation, with the expression of DAZL and DDX4 at culture Days 90–120 and an overall transcriptomic profile similar to mitotic pro-spermatogonia. Some cells reached the mitotic arrest pro-spermatogonia state. Global DNA methylation levels decreased to ∼10%.

hPGCLCs, human primordial germ cell-like cells; iMeLCs, incipient mesoderm-like cells; 4i, four inhibitors culture; Pre-ME, pre-mesendoderm precursors.

**Figure 2.**
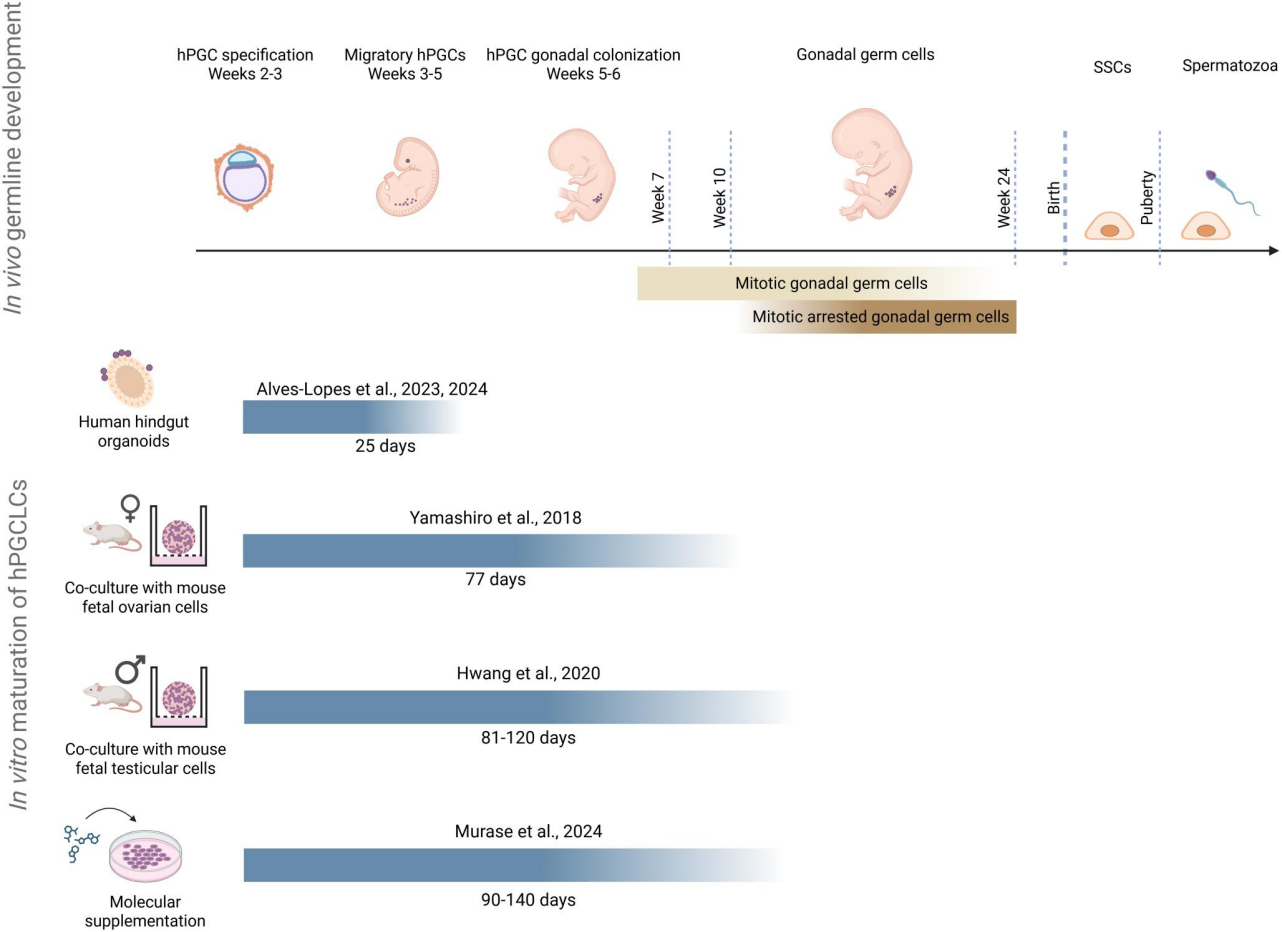
**Overview of current hPGCLC maturation protocols.** The upper panel illustrates the timeline of human male germline development, including embryonic and foetal stages, with mitotic gonadal germ cells and mitotic arrested gonadal germ cells spanning roughly from Weeks 5 to 24 and Weeks 10 to 24, respectively. The lower section illustrates four different approaches at maturing hPGCLCs, the culture periods, and the equivalent *in vivo* germ cell state achieved by each protocol. hPGCs, human primordial germ cells; SSCs, spermatogonial stem cells; hPGCLCs, human primordial germ cell-like cells. Figure created using BioRender (https://BioRender.com/w7z4vvs).

In their pioneer study, using iMeLCs as hPGCLC precursors (Sasaki *et al.*, 2015), Yamashiro *et al.* reported that, even in a sex-mismatched environment, male hPGCLCs cultured with ovarian somatic cells from mouse embryos can progress to mitotic gonocyte-like cells in 77 days (Fig. 2). These germ cells expressed DAZL and DDX4 and reached levels of DNA methylation close to 10%, mostly resembling embryonic mitotic gonocytes observed from Week 7 onwards (Li *et al.*, 2017; Yamashiro *et al.*, 2018). In a similar co-culture approach, male hPGCLCs aggregated with supporting testicular somatic cells from mouse embryos were able to progress up to a state of mitotic pro-spermatogonia within an 81-day timeframe (Hwang *et al.*, 2020) (Fig. 2). These germ cells showed similar evidence of DAZL and DDX4 expression and an overall transcriptomic signature resembling mitotic pro-spermatogonia (also known as mitotic gonocytes), observed in the human embryonic gonads from Week 7 onwards (Li *et al.*, 2017; Hwang *et al.*, 2020). However, most testicular tubular-like structures disaggregated by Day 80, thus causing the developing germ cells to lose contact with supporting Sertoli cells and limiting eventual further differentiation. Nevertheless, a small percentage of the remaining germ cells reached transcriptomic signatures similar to mitotic arrest pro-spermatogonia, observed in the human embryonic male gonads from Week 10 onwards (Fig. 2) (Li *et al.*, 2017; Hwang *et al.*, 2020).

Leveraging the differentiation potential of hPSCs into hindgut organoids, Alves-Lopes *et al.* (2023) developed a co-culture system that partially mimics the embryonic hindgut environment, which hPGCs traverse during migration. This system allowed hPGCLC maturation, with some cells expressing DAZL and DDX4 and progressively lowering genomic methylation markers after 25 days in culture (Fig. 2). Importantly, hPGCLCs derived from resetting precursors developed at a pace similar to that observed *in vivo*, whereas hPGCLCs derived from peri-gastrulation precursors demonstrated a slower progression potential. Establishing an hPGCLC maturation platform that relies exclusively on hPSCs provides an important step towards developing xeno-free and safer strategies for germline reconstitution.

In parallel with the efforts to mature hPGCLCs, identifying conditions that support proliferation, without losing their hPGC profile, could provide an important platform for increasing hPGCLC numbers, thus enabling downstream applications like progression assays and mechanistic analyses. Again, insights from murine studies were crucial and allowed the development of a medium supplemented with SCF, forskolin, and FGF2 that robustly propagates hPGCLCs for 30 days on mouse feeder cells (Murase *et al.*, 2020). Notably, hPGCLCs expanded under these conditions were reported to be more proliferative upon aggregation with ovarian somatic cells from mouse embryos than nascent hPGCLCs directly isolated from EBs (Murase *et al.*, 2020). More recently, a feeder-free alternative culture was reported to allow hPGCLC proliferation for at least 160 days, albeit still with the need to supplement the culture with conditioned media from mouse fibroblast cultures (Kobayashi *et al.*, 2022).

The conditions mentioned above supported hPGCLC propagation, yet with residual reconstitution of their embryonic progression. As such, there was a need to further characterize the dynamics involved in this process to achieve an ideal molecular supplementation-based system to recapitulate hPGCLC maturation. A key development in this sense was the finding that BMP proteins not only dictate hPGCLC specification but also regulate key aspects of their maturation (Murase *et al.*, 2024). While still relying on mouse feeder cells, Murase *et al.* demonstrated that BMP2 supplementation allowed the emergence of a significant portion of DAZL+/DDX4+ cells after 90 days of culture (Fig. 2). These hPGCLCs exhibited DNA demethylation at a slower rate than what happens *in vivo* but faster than in co-cultures of mouse embryonic somatic cells, achieving methylation levels as low as 10% that also extended to imprinted regions of the genome. According to the authors, hPGCLCs could be differentiated up to a mitotic pro-spermatogonia state, with a few germ cells reaching the mitotic arrest pro-spermatogonia state, making this the most efficient method to advance hPGCLCs *in vitro* to date.

Alternatively, gene overexpression approaches can offer a faster route for maturing hPGCLCs and serve as a model to explore the underlying mechanistic cues governing their development. Using male hESCs, Irie *et al.* (2023) expanded upon the role of DMRT1, known to be expressed in migratory hPGCs (Li *et al.*, 2017). Since the standard hPGCLC specification culture conditions do not support further maturation, they replaced the media after Day 2 with retinoic acid and Activin A supplementation. This switch led to a marked increase in the expression of migratory markers DMRT1 and CDH5 on Day 11 of culture, indicating an enhanced progression towards a more advanced state. Furthermore, when combining the media change with the simultaneous overexpression of DMRT1 and SOX17, the migratory/gonadal hPGC marker DAZL was expressed already at Day 3, further highlighting the role of DMRT1 in hPGCLC maturation, although genome-wide DNA demethylation was not detected (Irie *et al.*, 2023). Of note, female hiPSCs were reported to progress to oogonia-like cells within just 4 days following the overexpression of specific transcription factors (Pierson Smela *et al.*, 2025), a significant reduction compared to the typical 4-month timeline observed under somatic co-culture conditions (Yamashiro *et al.*, 2018).

The cumulative efforts in maturing male hPGCLCs are approaching comprehensive reconstitution of embryonic gametogenesis and have begun to recapitulate early foetal germ cell development (Table 2, Fig. 2). Given this, progressing hPGCLCs to the SSC state could become a feasible reality in a few years, opening the real chance of transplanting hiPSC-derived SSCs into infertile patients. With a perspective of ensuring that all germ cell states preceding SSCs are fully recapitulated and acquire the characteristics of their *in vivo* counterparts, a stepwise differentiation approach should emerge as the safer platform. Ideally, such an approach should also rely on a completely xeno-free and defined molecular-based culture. However, as noted in the prior studies, there is still a non-dispensable supportive role provided by somatic cells, either in 3D aggregates or as feeder cells.

## hiPSC generation from infertile (or at risk) male patients and germ cell fate specification

The growing recognition that hiPSC-based therapies may be the most viable option to restore the germline in certain populations of infertile male patients has motivated several research groups to establish hiPSC lines from individuals with compromised germ cell pools (Table 3). While most studies were limited to hiPSC generation and characterization (Kobayashi *et al.*, 2011; Zhou *et al.*, 2013; Shimizu *et al.*, 2016; Mouka *et al.*, 2017; Fang *et al.*, 2018; Luo *et al.*, 2018; Panula *et al.*, 2019; Alowaysi *et al.*, 2020; Wang *et al.*, 2020; Guo *et al.*, 2021; Luo *et al.*, 2021; Yan *et al.*, 2021; Gridina *et al.*, 2022; Harancher *et al.*, 2023; Gomez Limia *et al.*, 2025; Martin-Inaraja *et al.*, 2025), we direct our attention to those attempting subsequent germ cell differentiation (Ramathal *et al.*, 2014; Irie *et al.*, 2015; Zhao *et al.*, 2018; Wang *et al.*, 2019; Botman *et al.*, 2020; Fang *et al.*, 2020; Chang *et al.*, 2021; Abdyyev *et al.*, 2022; Mouka *et al.*, 2022; Esfahani *et al.*, 2024; He *et al.*, 2025). It is important to note that a significant proportion of these studies originated from patients with genetic abnormalities. As these persist in the respective hiPSC lines, their potential downstream applications may be conditioned. Nevertheless, it presents a great opportunity to consider the application of gene-correcting techniques (Xu *et al.*, 2017b; Cavaliere, 2018).

**Table 3.** hiPSC generation from male patients at risk of infertility and germline differentiation.

Reference	Cell source	Reprogramming method	hPGCLC specification	Outcome
Kobayashi *et al.*, 2011	Male patients undergoing treatment for infertility	Lentiviral delivery to testicular cells	No	Normal hPSC morphology; expression of pluripotency genes; differentiation into the three germ layers; 46, XY karyotype.
Zhou *et al.*, 2013	Male patient with cryptorchidism	Lentiviral delivery to urine cells	No	Normal hPSC morphology; expression of pluripotency genes; differentiation into the three germ layers; 46, XY karyotype.
Shimizu *et al.*, 2016	Male patient with Klinefelter syndrome (47, XXY)	Sendai viral delivery to testicular fibroblasts	No	Normal hPSC morphology; expression of pluripotency genes; differentiation into the three germ layers; 47, XXY karyotype.
Mouka *et al.*, 2017	Male patient with azoospermia (aberrations on chromosomes 7 and 12)	Sendai viral delivery to erythroblasts	No	Normal hPSC morphology; expression of pluripotency genes; differentiation into the three germ layers; 46, XY karyotype with original aberrations on Chromosomes 7 and 12.
Luo *et al.*, 2018	Male patient with Turner syndrome (45, X/46, XY mosaicism)	Sendai viral delivery to peripheral blood mononuclear cells	No	Normal hPSC morphology; expression of pluripotency genes; differentiation into the three germ layers; original 45, X/46, XY karyotype.
Fang *et al.*, 2018	Male patient with idiopathic infertility	Retroviral delivery to foreskin fibroblasts	No	Normal hPSC morphology; expression of pluripotency genes; differentiation into the three germ layers; 46, XY karyotype.
Panula *et al.*, 2019	Male patients with Klinefelter syndrome (47, XXY)	Episomal vector delivery to dermal fibroblasts	No	Normal hPSC morphology; expression of pluripotency genes; differentiation into the three germ layers; 47, XXY karyotype. Differentially expressed genes related to fertility when compared to hiPSCs from healthy controls.
Wang *et al.*, 2020	Male patient with Sertoli cell only syndrome and c.731_732delAT mutation in the PIWIL2 gene.	Retroviral delivery to dermal fibroblasts.	No	Normal hPSC morphology; expression of pluripotency genes; differentiation into the three germ layers; 46, XY karyotype with c.731_732delAT mutation in the PIWIL2 gene.
Alowaysi *et al.*, 2020	Male patient with Klinefelter syndrome (49, XXXXY).	Non-modified synthetic mRNA delivery to dermal fibroblasts.	No	Normal hPSC morphology; expression of pluripotency genes; differentiation into the three germ layers; 49, XXXXY karyotype.
Yan *et al.*, 2021	Male patient with SRY-positive 46, XX testicular disorder of sex development.	Sendai viral delivery to peripheral blood mononuclear cells.	No	Normal hPSC morphology; expression of pluripotency genes; differentiation into the three germ layers; 46, XX karyotype.
Guo *et al.*, 2021	Male azoospermic patient with translocation between Chromosomes 13 and 14.	Episomal vector delivery to peripheral blood mononuclear cells.	No	Normal hPSC morphology; expression of pluripotency genes; differentiation into the three germ layers; karyotype with translocation between Chromosomes 13 and 14.
Luo *et al.*, 2021	Male patient with SRY-positive 46, XX testicular disorder of sex development.	Sendai viral delivery to peripheral blood mononucle.ar cells	No	Normal hPSC morphology; expression of pluripotency genes; differentiation into the three germ layers; 46, XX karyotype.
Gridina *et al.*, 2022	Male patient with small supernumerary marker Chromosome 4.	Episomal vector delivery to dermal fibroblasts.	No	Normal hPSC morphology; expression of pluripotency genes; differentiation into the three germ layers; 47, XY, +r(4) karyotype.
Harancher *et al.*, 2023	Male patient with sickle cell disease	Sendai viral delivery to testicular fibroblasts	No	Normal hPSC morphology; expression of pluripotency genes; differentiation into the three germ layers; 46, XY karyotype.
Martin-Inaraja *et al.*, 2025	Male patient with Klinefelter syndrome (47, XXY)	Sendai viral delivery to testicular somatic cells	No	Normal hPSC morphology; expression of pluripotency genes; differentiation into the three germ layers; 47, XXY karyotype.
Gomez Limia *et al.*, 2025	Male patient with prune belly syndrome	Sendai viral delivery to dermal fibroblasts	No	Normal hPSC morphology; expression of pluripotency genes; differentiation into the three germ layers; 46, XY karyotype.
Ramathal *et al.*, 2014	Male patients with azoospermia	Lentiviral and mRNA delivery to dermal fibroblasts	Undefined Transplantation of hiPSCs into murine seminiferous tubules	Reduced germ cell competence relative to healthy hPSCs.
Zhao *et al.*, 2018	Male patients with non-obstructive azoospermia (two with Sertoli-cell only syndrome and two with microdeletions in the AZFc locus)	Retroviral and episomal vector delivery to dermal fibroblasts	Undefined Chemically defined differentiation media	Reduced germ cell competence from Sertoli-cell only syndrome hiPSCs relative to healthy hPSCs. Comparable germ cell competence between hiPSCs with AZFc deletions and healthy hPSCs.
Botman *et al.*, 2020	Male patient with Klinefelter syndrome (47, XXY)	Lentiviral delivery to dermal fibroblasts	Undefined Differentiation media supplemented with BMP4, GDNF, retinoic acid and SCF	Compromised germ cell competence. Higher apoptosis during differentiation.
Irie *et al.*, 2015	Male patient with Fragile X syndrome	N/A	Defined via peri-gastrulation precursors	Comparable hPGCLC specification efficacy relative to healthy hPSCs.
Wang *et al.*, 2019	Male patient with obstructive azoospermia	Episomal vector delivery to testicular cells	Defined via peri-gastrulation precursors	Comparable hPGCLC specification efficacy relative to healthy hPSCs.
Fang *et al.*, 2020	Male patients with non-obstructive azoospermia	Retroviral delivery to dermal fibroblasts	Defined via peri-gastrulation precursors	Normal hPSC morphology; expression of pluripotency genes; differentiation into the three germ layers; 46, XY karyotype. Compromised germ cell competence relative to healthy hPSCs. Higher apoptosis.
Chang *et al.*, 2021	Male patients with non-obstructive azoospermia	Sendai viral delivery to peripheral blood mononuclear cells	Defined via peri-gastrulation precursors	Lower expression of hPGCLC markers PRDM1 and STELLA in patient-derived hPGCLCs.
Abdyyev *et al.*, 2022	Male patient with Down syndrome (47, XY, + 21(20))	Episomal vector delivery to dermal fibroblasts	Defined via peri-gastrulation precursors	Compromised germ cell competence relative to healthy hPSCs.
Mouka *et al.*, 2022	Male patient with non-obstructive azoospermia. Patient from consanguineous family.	Sendai viral delivery to erythroblasts	Defined via peri-gastrulation precursors	Compromised germ cell competence relative to healthy hPSCs.
Esfahani *et al.*, 2024	Male patients with non-obstructive azoospermia.	Sendai viral delivery to dermal fibroblasts	Defined directly via primed hPSCs	Retained capability for germ cell competence.
He *et al.*, 2025	Male patients with Klinefelter syndrome	Episomal vector delivery to dermal fibroblasts	Defined via peri-gastrulation precursors	Compromised germ cell competence relative to healthy hPSCs.

hPGCLCs, human primordial germ cell-like cells; hPSCs, human pluripotent stem cells; hiPSCs, human-induced pluripotent stem cells.

Genetic syndromes affecting sex chromosomes represent a frequent cause of male infertility (Krausz and Riera-Escamilla, 2018). Some reports of germ cell differentiation from hiPSCs of such patients still relied on undefined protocols to obtain germ cells (Ramathal *et al.*, 2014; Zhao *et al.*, 2018; Botman *et al.*, 2020). Nevertheless, recent studies hinted that genetic abnormalities associated with low physiological sperm production may impair the germline already at the embryonic stage, as evidenced by lower hPGCLC specification efficacies (Fang *et al.*, 2020; Abdyyev *et al.*, 2022; He *et al.*, 2025).

Ramathal *et al.* (2014) explored germ cell differentiation from hiPSCs derived from azoospermic patients carrying deletions in the AZF region of the Y chromosome, one of the most common genetic causes of male infertility (Rabinowitz *et al.*, 2021). Despite showing some capacity for germline differentiation under BMP-supplemented monolayer conditions, all patient-derived lines exhibited a markedly reduced efficiency compared to control hiPSCs. Among them, lines with the AZFa and AZFb deletions exhibited a reduced competence for germ cell differentiation compared to those with AZFc deletions, reflecting their poorer clinical prognosis of sperm extraction (Hopps *et al.*, 2003). In another study, Zhao *et al.* (2018) examined germ cell differentiation from hiPSCs derived from patients with AZFc deletions and Sertoli cell-only syndrome, with both exhibiting a reduced capacity to initiate germline differentiation. Concerning sex chromosome aneuploidies, hiPSCs from a Klinefelter syndrome patient were reported to yield a lower fraction of differentiated germ cells relative to healthy controls, which was hypothesized to be due to an increase in apoptosis conferred by the extra copy of the X chromosome (Botman *et al.*, 2020).

In addition to previous reports attempting differentiation of germ cells using undefined protocols, there were several attempts to obtain hPGCLCs from hiPSCs of infertile patients using defined methods. Irie *et al.* (2015) demonstrated comparable TNAP+/CD38+ hPGCLC yields across different healthy hESC/hiPSC lines and a male fragile X syndrome hiPSC line after 4 days under specification conditions. Similarly, hiPSCs derived from a patient with obstructive azoospermia could specify EPCAM+/ITA6+ hPGCLCs via iMeLCs, after 4–8 days under specification conditions, and resume normal epigenetic reprogramming, as assessed by an increase in the intermediary demethylation marker 5hmC (Wang *et al.*, 2019). Furthermore, another study obtained hPGCLCs from 4i-cultured hiPSCs of a patient bearing a rearrangement between Chromosomes 7 and 12 and a patient from a consanguineous family, as demonstrated by comparable TFAP2C and PDPN marker expression to controls after 4 days under specification conditions (Mouka *et al.*, 2022).

Conversely, it was also evident that some patient-derived hiPSCs are compromised in their ability to generate hPGCLCs. Using hiPSCs from idiopathic non-obstructive azoospermia patients and the iMeLC protocol, Fang *et al.* (2020) demonstrated that there was a reduced specification efficiency of EPCAM+/ITA6+ hPGCLCs and increased apoptosis rates at Day 4 relative to hPGCLCs specified from healthy hiPSCs. Likewise, Chang *et al.* (2021) noted that, while non-obstructive azoospermia patient-derived hiPSCs were competent for hPGCLC specification using the 4i protocol, the expression of hPGCLC markers *PRDM1* and *DPPA3* was reduced at specification Day 4 relative to healthy controls.

More recently, a mechanistic study expanded upon the genomic dysregulation responsible for the lower hPGCLC specification potential in hiPSCs derived from Klinefelter syndrome patients (He *et al.*, 2025). After 4 days of specification using the iMeLC protocol, the authors reported the emergence of very few EPCAM+/ITA6+ hPGCLCs relative to healthy hiPSCs, which was explained to be the result of an imbalance in the X-linked gene dosage. Accordingly, one of the escapee genes was found to upregulate *SOX2* expression, which in turn increases the ectoderm fate while inhibiting mesendoderm commitment.

While not as well-characterized, some non-sex chromosome aberrations are also associated with male infertility (Marzouni *et al.*, 2021). Building upon the notion that Down syndrome patients are admitted to be infertile (Parizot *et al.*, 2019), Abdyyev *et al.* (2022) pointed out that germline commitment appeared to be impaired as early as the hPGC specification state. Using a peri-gastrulation precursor-based protocol, the authors noted a significant reduction in the specification efficacy of SSEA1+ hPGCLCs at Day 6 relative to a healthy control male line. In addition, key early hPGCLC regulator genes *PRDM14* and *SOX17*, as well as the cell-surface marker CD38, were shown to be downregulated on Day 6 of specification. This points to a remarkable potential for patient-derived hiPSCs, which can be used not only with clinical applications in mind but also as a tool to model reproductive pathologies.

In line with the trend to switch to higher-yielding protocols to specify hPGCLCs, Esfahani *et al.* (2024) screened the germline competence of eight non-obstructive azoospermia patient-derived hiPSCs using their gel-3D culture. Interestingly, all lines seemed competent for the generation of hPGCLCs expressing TFAP2C, SOX17, and NANOG after a specification culture of 8 days, though the genetic background of the patients was not detailed.

As summarized in Table 3, several publications have demonstrated the feasibility of generating hiPSC lines from infertile patients and differentiating them in the germline fate, albeit with varying degrees of success. However, a key aspect that remains largely unexplored is the maturation potential of hPGCLCs obtained from these patient-specific hiPSCs.

It is evident that some infertility-causing conditions have a genetic basis that hinders germline commitment already at the embryonic stage. As a result, hiPSCs derived from these patients are expected to bear this genomic signature and exhibit impaired competence for hPGCLC specification. However, the existence of genetic mosaicism, such as in patients with Klinefelter syndrome (Garcia-Quevedo *et al.*, 2011), raises the possibility of collecting somatic cells with a healthy genotype. In addition, if a genetic abnormality known to cause infertility is identified in hiPSCs, there are already established techniques that can be used to amend them. For instance, the characteristic gene mutation responsible for Huntington disease was corrected in patient-hiPSCs using CRISPR/Cas9, thus allowing their successful differentiation into healthy neurons (Xu *et al.*, 2017a). Moreover, even large-scale chromosomal aneuploidies, such as the extra Chromosome 21 in Down syndrome, have been experimentally reversed in hiPSCs (Hashizume *et al.*, 2025).

Importantly, infertile cancer survivors comprise one of the main groups that can benefit from hiPSC technologies and, in particular, hiPSC-derived SSCs. However, we have noted a significant lack of hiPSC generation and hPGCLC specification studies from these patients. Nevertheless, in a sporadic study, a viral-based hiPSC generation from infertile subjects was performed using fresh non-tumoural testicular tissue from a testicular cancer patient (Kobayashi *et al.*, 2011). There is also a report of hiPSC generation using a viral reprogramming protocol of testicular fibroblasts extracted from the cryopreserved tissue sample of a sickle cell disease patient (Harancher *et al.*, 2023). Accordingly, this opens the possibility of using cryopreserved testicular samples collected prior to therapy (Duffin *et al.*, 2024) for hiPSC generation and subsequent use for regenerative purposes to address side effect problems caused by chemotherapy or radiotherapy, including infertility.

## The promises and challenges for clinical applications

The significant strides in *in vitro* gametogenesis achieved over the past decade highlight the increasing prospects of clinical applications. Despite still considering the use of hPSC-derived germ cells premature in their last 2021 guideline revision, the International Society for Stem Cell Research (ISSCR) further noted its feasibility after sufficient safety, policy, and regulatory concerns are dealt with (International Society for Stem Cell Research, 2021).

From our perspective, this prompts two main concerns that should be addressed in the effort to narrow the bridge between research and clinical applications. First, there needs to be a stronger emphasis on developing defined protocols for hPGCLC specification and maturation that comply with good manufacturing practices (GMPs). Second, and most importantly, *in vitro*-derived SSCs would need to be assessed with strict safety parameters, both prior to and after their transplantation in the patients’ testes. In the event of the successful *in vitro* recapitulation of the spermatogenesis process, the safety evaluation of hiPSC-derived sperm may even involve *in vitro* fertilization and embryo cultures, likely igniting a fierce ethical debate akin to what is already actively discussed for embryo models (Rivron *et al.*, 2023; Mallapaty, 2024).

Indeed, performing biochemical assays on hiPSC-derived SSCs and sperm can rule out major contamination, genetic and epigenetic abnormalities, but the ultimate analysis of their functionality requires fertilization and subsequent embryo development, perhaps even beyond the broadly accepted ‘14-day rule’ (Appleby and Bredenoord, 2018). Therefore, there is an urgent need to raise awareness and discussion among the public, policymakers, and stakeholders on the ultimate beneficence principles that should guide research in this field.

### Compliant good manufacturing practices and safety

As noted in Table 3, the main challenges in transposing much of the described efforts to obtain hiPSC lines and specifying and maturing hPGCLCs are the strong reliance on genome-integrating reprogramming vectors and animal-derived cell culture components. While some of the gathered studies have used safer non-integrating RNA- and episomal-based reprogramming methods, these are known to have lower efficiency, potentially increasing the burden of reliably obtaining viable stem clones (Bailly *et al.*, 2022). In an effort to tackle these concerns, the emergence of chemical-based reprogramming strategies, whereby the pathways governing pluripotency are activated without exogenous gene delivery into somatic cells, is becoming an increasingly popular alternative (Wen *et al.*, 2025). Of note, a recently developed protocol managed to achieve a remarkable efficiency of up to ∼30% in hiPSC generation from mesenchymal stromal cells and dermal fibroblasts (Liuyang *et al.*, 2023). In addition, there are hints from the mouse model that chemically reprogrammed iPSCs have a closer epigenetic profile to mESCs than iPSCs obtained after the retroviral delivery of the Yamanaka factors (Ping *et al.*, 2018). As such, evidence is pointing towards chemical reprogramming, enabling a safer, more standardized, and cost-effective approach.

Another common concern regarding hiPSC generation is the association between the core pluripotency network and potential oncogenic pathways, specifically with the induction of the *MYC* factor and its downstream effectors (Lee *et al.*, 2013). Genetic abnormalities resulting from the reprogramming process and hiPSC culturing constitute another valid worry in the application of hiPSC-based therapies (Assou *et al.*, 2020; Poetsch *et al.*, 2022). However, given that these mutations are bound to accumulate over time in hiPSC cultures (Kuijk *et al.*, 2020), this concern can be mitigated by using freshly derived hiPSCs for clinical applications. Indeed, as most ongoing clinical trials must follow strict safety rules, no imminent adverse events regarding tumorigenicity and mutations are apparent (Kelly *et al.*, 2024; Sawamoto *et al.*, 2025).

Parallel to these considerations, hiPSC maintenance was not always carried out in chemically defined, xeno-free conditions. Initially, most cultures relied on foetal bovine serum (FBS) supplementation as the source of nutrients for cellular metabolism and growth. FBS has an undefined composition and batch-to-batch variability while also bearing the threat of containing xenogeneic antigens and becoming a source of zoonotic infections (Subbiahanadar Chelladurai *et al.*, 2021). Likewise, these concerns extend to commonly used feeder cells and coating substrate matrices needed for the maintenance of hiPSC pluripotency (e.g. mouse embryonic fibroblasts and Matrigel) (Al Abbar *et al.*, 2020). Nonetheless, the use of chemically defined and animal-free cultures is the current standard in the stem cell field, owing to the development of multiple xeno-free protocols over the years, as reviewed previously (Liu *et al.*, 2020).

Achieving efficient hiPSC differentiation and tissue-specific organization in similar xeno-free conditions is critical for clinical applications. A growing number of protocols have demonstrated their feasibility across multiple somatic cell fates, including the generation of hepatocytes (Vanmarcke *et al.*, 2023) and neural cells (Rust *et al.*, 2022). Currently, there are efforts to transpose this notion in the field of germline reconstitution, both with the feeder-free support of hPGCLC proliferation (Kobayashi *et al.*, 2022) and molecular supplementation-based maturation attempts (Murase *et al.*, 2024).

Despite these concerns, several ongoing hiPSC clinical trials were approved while still relying on animal-derived material. For instance, the generation of retinal cell sheet structures for the treatment of macular degeneration uses a porcine-derived scaffold structure to allow correct cell assembly (Mandai *et al.*, 2017). The multitude of safety analyses performed included an adhesion test to rule out trans-differentiated cells, identification of human and animal pathogens, whole-genome sequencing, DNA methylation analysis, and tumorigenicity assays. Another example is the supplementation with FBS on a cardiomyocyte differentiation culture for the treatment of ischaemic cardiomyopathy (Miyagawa *et al.*, 2022). Likewise, the pre-clinical safety assessment was comprehensive and worthy of its own separate publication (Miyagawa *et al.*, 2024). Admittedly, with the best interest of the patients and their progeny in mind, a more lenient attitude towards a fully xeno-free environment can be justified, based on extensive safety testing. Reflecting the need for rigorous oversight, the ISSCR has recently outlined six key areas for quality testing in pluripotent stem cell-derived therapies: cell type-specific testing, genomic stability, safety, functional and epigenetic testing, and ensuring the consistency of cell-handling protocols (International Society for Stem Cell Research, 2025).

### Assessing the genetic and epigenetic profiles of *in vitro*-derived germ cells

Maintaining a genetic and epigenetic profile that mirrors its physiological counterparts is crucial for proper cell functionality and a paramount aspect of ART (Mani *et al.*, 2020). Therefore, we believe there are two crucial points worth discussing regarding the role of epigenetics in safety evaluations.

First, as noted in the introduction section, reprogramming somatic cells to primed hiPSCs involves an extensive epigenome remodelling that, if incomplete, may result in a retained ‘epigenetic memory’ that compromises downstream differentiation attempts (Kim *et al.*, 2010). An ingenious approach to erasing these residual characteristics involves converting hiPSCs to the naïve state of pluripotency, achieving global DNA hypomethylation, and then reverting them back to primed pluripotency, thereby allowing the establishment of a correct methylation pattern. However, some protocols for naïve conversion were shown to also induce the demethylation of imprinted regions of the genome (Keshet and Benvenisty, 2021). Given that these are not restored upon culturing in primed conditions, there is an ongoing push in the field to refine naïve culture protocols and avoid imprint erasure (De Los Angeles, 2025), such as strategies that slightly tweak the naïve culture composition to shorten the overall conversion timeframe (Buckberry *et al.*, 2023).

The second moment where epigenetic validation assumes a critical role is in the assessment of the epigenetic profiles throughout hPGCLC specification and maturation. Upon hPGC specification and migration, there is a progressive decrease in global DNA methylation to basal levels as low as 5% as hPGCs settle in the developing gonads (Lee and Surani, 2024). This demethylation programme extends to imprinted regions of the genome, which were shown to start hypomethylation upon gonad colonization (von Meyenn and Reik, 2015). This extensive DNA demethylation is a hallmark of epigenetic reprogramming in the human germline, ensuring the erasure of parental epigenetic memory.

Following the extensive genome-wide demethylation, an equally critical process of DNA remethylation occurs in a sex-specific manner. In males, *de novo* DNA methylation is initiated during the late foetal period, likely in the third trimester, and is largely completed postnatally, resulting in high levels of DNA methylation in mature pro-spermatogonia (Li *et al.*, 2021). In contrast, female germ cells remain globally hypomethylated until after birth, with DNA remethylation occurring progressively during oocyte growth and maturation (Anvar *et al.*, 2021). Importantly, this process also involves the re-establishment of methylation at imprinting control regions (ICRs), which are methylated in a parent-of-origin-specific manner to ensure proper monoallelic expression of imprinted genes. The precise timing and fidelity of the remethylation phase (also known as the programming phase) are essential for germ cell competence and the epigenetic integrity of the next generation. Furthermore, achieving complete epigenetic reconstitution at this state and clearance of abnormal cells is a critical safety aspect, particularly given that seminomas can arise from globally hypomethylated hPGCs (Cheng *et al.*, 2024). Currently, while hPGCLC maturation strategies hint at a successful reconstitution of the DNA demethylation stage (Yamashiro *et al.*, 2018; Hwang *et al.*, 2020; Murase *et al.*, 2024), no protocol to date has pushed them further to the DNA programming phase.

Beyond genome-wide changes in DNA methylation levels, there are several other known epigenetic modifications driving hPGC maturation. Histone modifications, in particular, offer an additional layer of transcriptional regulation, such as the concomitant loss of the repressive mark H3K27me3 and the increase in the active mark H3K9ac during the first trimester (Ramakrishna *et al.*, 2021; Gruhn *et al.*, 2023).

Currently, there are still some gaps in the characterization of the epigenetic landscape of nascent hPGCs (Weeks 2–6) and the maturing foetal germ cells (second and third trimester), owing to the restricted access to human embryos and foetuses and the low quantities of starting biological material (Ramakrishna *et al.*, 2021; Rugg-Gunn *et al.*, 2023). Therefore, the development of more sensitive techniques is key for unravelling hPGC physiological epigenetic features that can then be used to validate hPGCLC maturation. Recently, the development of a target chromatin indexing and tagmentation method allowed the profiling of histone modifications at the single-cell level, shedding light on regulatory elements and transcription factors involved in mouse embryo development from the stage of zygote to blastocyst (Liu *et al.*, 2025). Additionally, physiological SSCs also lack comprehensive epigenetic profiling, which is essential for benchmarking eventual hiPSC-derived SSCs, though this need has deserved increased recognition (Cheng and McCarrey, 2023). As an example, emerging evidence from the mouse model demonstrates that epigenetics plays a critical role in regulating the self-renewing and differentiation-priming capabilities of SSCs (Cheng *et al.*, 2020). Therefore, reconstituting the epigenetic profile of hiPSC-derived SSCs is critical to ensuring their functional fidelity and, ultimately, positive clinical outcomes in infertility treatments.

Taken together, the rapidly evolving strides in mapping epigenetic features will likely contribute to a more comprehensive characterization of hiPSC-derived SSCs and sperm as an important layer of safety in guaranteeing their functional resemblance to their *in vivo* counterparts.

### Ethical, social, and political landscape

Given the immense possibilities and the sheer number of individuals potentially benefiting from hiPSC-derived SSCs (Le Goff *et al.*, 2024), it is our belief that their generation and clinical application are not just possible but also a close future reality. The achievements of the past years indicate that we are swiftly advancing towards a future where patient-specific germline regeneration will become a key tool in reproductive medicine. Although full germline reconstitution in the mouse model was rapidly achieved from the description of iPSCs around 2006 (Takahashi and Yamanaka, 2006) to the generation of viable iPSC-derived offspring in 2011 (Hayashi *et al.*, 2011, 2012), the respective human counterpart is also moving at a prompt, yet cautious pace. As such, it would be advisable to establish early conceptual frameworks for integrating *in vitro* gametogenesis into the range of options offered by ARTs, to facilitate a smooth transition in the future.

In this sense, Galea (2021) asserts that most negative opinions towards *in vitro* gametogenesis stem from the safety point of view and deconstructs the most common objections against its use in the clinic. The author argues that concerns about new kinds of parenthood are not fundamentally different from those provided by existing fertility treatments, and that objections based on embryo farming or ‘designer babies’ are issues of regulation rather than inherent to *in vitro* gametogenesis itself. In addition, the ‘slippery slope’ argument regarding the subsequent approval of human cloning is also dismissed, and objections based on *in vitro* gametogenesis being ‘unnatural’ could also be applied to most medical interventions.

While currently there are no regulations pertaining specifically to the implementation of *in vitro* gametogenesis, several events and campaigns to raise awareness are being held throughout the world. In the USA, the regulatory framework of *in vitro* gametogenesis will likely be under the domain of the US Food and Drug Administration (FDA). Accordingly, Peter Marks, former director of the Center for Biologics Evaluation and Research at the FDA, highlighted three main considerations that should be addressed during preclinical research that generates embryos from hiPSC-derived gametes. Two of them pertain to genomic integrity, both from the perspective of ruling out any sort of abnormality, but also confirming genomic stability throughout multiple generations in animal models. In addition, the importance of establishing safeguards to prevent the potential misuse of this technology was also emphasized (National Academies of Sciences, Engineering, and Medicine, 2023). In a conference held at Lancaster University, Emily Jackson, Professor of Law at the London School of Economics and Political Science, raised relevant considerations regarding the country’s legal parenthood recognition, especially if male hiPSCs can be differentiated in gametes from both sexes (Bolton, 2023). Despite the novel opportunities offered by hiPSC-derived SSCs and sperm, conventional ART for fertility treatments still need substantial policy-wise improvements to ensure universal, equitable access and general public education (Fertility Europe, 2024).

In this sense, there is an additional concern regarding the research of hiPSC-derived SSCs and sperm. While traditional ARTs were developed using mostly public funding and remained free of intellectual claims, there is the risk that technologies involving hiPSC-derived SSCs and sperm may have certain components patented owing to private investment and the complex nature of the procedures, potentially restricting their equitable use (Cyranoski *et al.*, 2023). If we momentarily shift our attention to the field of organoid modelling, in which the correct reconstruction of intricate tissues is imperative, many patent applications have already been filed across different jurisdictions (Zhu *et al.*, 2022). These include not only culture media and supporting growth materials/devices but also encompass the downstream applications of the organoid models. As such, we extend our hopes that regulatory agencies foster a cooperative environment between research universities and business stakeholders, ensuring fair and open access to hiPSC technologies (Le Goff *et al.*, 2024).

Given the myriad applications offered by hiPSC-derived SSCs and sperm, from more direct applications in reproductive medicine to modelling human germline development and drug screening, their transformative potential warrants strategic governmental and private investment in the field.

## Discussion

In recent years, stem cell technologies have emerged as one of the most transformative tools in regenerative medicine, offering possibilities for restoring patients’ impaired tissues and organ function. In the clinical setting, the use of hPSC-derived therapies is already a reality that addresses a growing number of conditions (Kirkeby *et al.*, 2025). Among these, it is our conviction that the restoration of the human germline in infertile patients will not be an exception. In fact, it represents one of the most compelling applications of these technologies.

Over the past decade, remarkable scientific advances have pushed boundaries in the field of *in vitro* human male gametogenesis, especially with the development of robust protocols for hPGCLC specification and maturation, modelling the early embryonic steps of germline development. Subsequent maturation protocols still need some work in pushing hPGCLCs up to the SSC state. For many patients, particularly those with a completely depleted germline due to medical treatments or genetic conditions, hiPSC-derived SSCs and sperm may eventually offer the most viable path to biological parenthood.

In an effort to expand current experimental fertility preservation strategies for prepubertal patients undergoing potentially gonadotoxic therapies (Duffin *et al.*, 2024), we propose leveraging the expanding biobank of patient-derived testicular tissues as an already established resource for patient-specific hiPSCs and subsequent germline restoration. As such, clinicians should be equipped to inform patients not only about current fertility preservation options but also about the evolving landscape of regenerative reproductive technologies.

In addition to addressing cases of infertility, there are several other less evident advantages stemming from the generation of hiPSC-derived SSCs and sperm. For instance, being able to generate gametes on an as-needed basis would not only eliminate the need for long-term storage but also solve risks and inconveniences arising from gamete donation (Cattapan, 2016). From the perspective of expanding reproductive possibilities, there is even a significant potential for same-sex couples to have genetically related offspring (Murakami *et al.*, 2023). Yet, realizing this potential requires overcoming critical hurdles, as also mentioned by the authors. Ensuring the maintenance of the characteristic female epigenetic landscape in male cell-derived oocytes, mitigating the risks of genetic and epigenetic errors during prolonged *in vitro* culture, and addressing the low embryo viability, where only 1% of the implanted embryos progressed to a full-term pregnancy, are some of the key challenges to consider before starting the translation of the technology to the human context (Bayerl and Laird, 2023; Ledford and Kozlov, 2023).

hiPSC-derived SSCs and sperm can also serve as a critical tool in addressing future global health challenges. Fertility rates are already declining across many developed regions of the globe, and the climate-change-driven spread of infectious diseases and novel environmental threats (Semenza *et al.*, 2022), many of which with yet uncharacterized effects on fertility, adds further concern. Emerging pathogens such as Zika virus (Almeida *et al.*, 2020), Ebola (Coffin *et al.*, 2018), and many other viral, bacterial, and parasitic infections (Guiton and Drevet, 2023) have been linked to potential prolonged adverse effects on male fertility. Moreover, the growing awareness of microplastic accumulation across several organs, including the testes (Zhao *et al.*, 2023; Hu *et al.*, 2024), and their resulting gonadotoxic effects further underscores the need for innovative reproductive technologies that preserve and restore fertility.

## Conclusion

Moving forward, the field should focus on the refinement of maturation protocols, rigorous safety assessments, and the development of standardized laboratory practices to ensure reproducibility and minimize risk. In parallel, discussions surrounding the ethical, legal, and social implications of human germline regeneration must shift towards a more mainstream audience, ensuring the appropriate regulatory frameworks for its responsible access. As we look ahead, the reconstitution of the human male germline from hiPSCs would constitute one of the major breakthroughs in reproductive medicine, not only ultimately providing infertile patients with hope for biological parenthood but also expanding overall reproductive possibilities.

## Data Availability

No new data were generated or analysed in support of this research.
